# Multiple facets of learning a skill – Amalgamation of learning theories in cadaveric surgical skill lab 

**DOI:** 10.30476/jamp.2020.84959.1155

**Published:** 2021-07

**Authors:** DINESH KUMAR. V, RAJASEKHAR S.S.S.N

**Affiliations:** 1 Department of Anatomy, Jawaharlal Institute of Postgraduate Medical Education and Research, Puducherry-605006, India

## Dear Editor

Psychomotor skills training includes three stages of learning. In the cognitive stage, there is a creation of awareness regarding the items to be learned; in the associative stage, the refining of skills takes place, and in the autonomous stage, the proficiency in the skills is developed ( 1 ). One of the ways by which novice surgeons acquire mastery standards, before proceeding to perform surgical skills independently, is by practicing in cadavers. In contrast to the conventional pattern of learning, i.e. apprenticeship model whereby the trainees learn by ‘see one, do one and teach one’ adage, cadaveric skill lab ascertains the transformation/change of individual abilities in a structured environment in pursuit of a task goal. The above statement could be substantiated by the study conducted by Zendejas et al. ( 2 ), where residents trained under simulated settings were able to complete the task goal, i.e. total extra-peritoneal repair in lesser time and with reduced intra-operative and post-operative complications compared to the traditional training group.

When we mean acquiring mastery standards, it means that all learners should attain the prescribed competency standards required for deliberate practice without much variation in the defined outcomes. Nevertheless, it is well known that each individual does not necessarily have the same motor learning dynamics, and they need not learn the same pattern even in similar task conditions ( 3 ). Notably, the surgical skill can be deciphered as a sequential motor skill learning, involving a combination of explicit and implicit processes, operating in a schema in order to accomplish the task ( 4 ). Besides, this should be complemented by proprioceptive feedback, which harnesses the motor accuracy and enables making quick adjustments in the authentic environment. In this context, we wish to throw light upon two pertinent learning theories which medical educators shall reconcile while planning surgical skill programs.

Let us consider the sequence of learning taking place while learning a surgical skill, for example, arthrocentesis in the cadaveric knee model. Comparing the *Kovacs’ steps for procedural skill training* ( 5 ), we could envisage that skill acquisition passes through four distinct stages. In the first step, the novice gains the cognitive knowledge required for the procedure (‘learn’ step). Secondly, he/she visualizes and contextualizes the procedure by observing while being performed by an expert (‘see’ step). Thirdly, the step of imitation begins where the participant performs the movements required for the skill with a certain degree of confidence, and this could be considered as trial and error (‘practice’ step). Finally, upon multiple cycles of practice, the trainee would master the movement patterns, tailor it according to tricky situations, and, if needed, create a new pattern of movements (‘do’ step). Subsequently, we shall reiterate upon two theories, which could be attributed to motor learning in the cadaveric skill lab.

Schmidt’s schema theory is an open-loop controlled method of motor learning which does not involve feedback mechanism ( 6 ). It postulates the mechanism by which a novice learns a specific motor task which he or she has not performed previously. During the ‘learn’ and ‘see’ phase, a trainee observes a lot of co-ordinated movement patterns that get stored as schemas in a short memory system. When they practice, they learn by committing multiple errors, which also gets coded as schemas. For example, while performing laparoscopic procedures in cadavers, a surgeon needs to learn how to anticipate where and how far the instrument will pass through employing perceptual decisions made on the moment-moment basis. From short term memory, the information goes to a recall schema, where the specific response is selected, and then the recognition schema, where the response will be evaluated, errors are realized, and followed by the generation of information on the correct response. This process helps in deciding a critical move or use a certain degree of force in specific situations for a co-ordinated motor pattern. This can be analogized to a cricketer who recalls the mistakes after the innings, interprets the sequence of movements, and corrects his future performances. Besides, schema theory emphasizes the value of variability in practice conditions for honing the learned skills in simulated settings. Thus, schema theory could still be considered to have a significant role in skill learning despite the criticism placed on it.

Another major theory that has been posited for motor skill acquisition is Adam’s theory of learning, which is essentially a *closed-loop* method relying upon the sensory/perceptual feedback on the ongoing sequence of skilled movements ( 7 ). It involves a stimulus (input) that generates a perception of input resulting in a memory trace to decide as to which movement should be initiated. This will be followed by a perception trace, which is an amalgamation of sensory consequences of previous similar movements and, finally, a motor action (output). This can be compared with gaining experience of driving a car in crowded places where the driver learns negotiation and adjustment abilities in stimulus-response reacting manner. The crux of this theory that supports the need for formative feedback in ‘practice’ sessions whereby making the learners know about the results is that it facilitates the identification/rectification of errors. To exemplify, a workshop where the placement of the endotracheal tube in cadaver by the novice is confirmed by visualizing using the ultrasound transducer, the learners could assess the end-point by themselves and rectify accordingly. Performing the prescribed movement repetitively enables the learners to gain the correct perceptual trace over time with the actual accomplishment of end-point, similar to hitting the bull’s eye during shooting practice. Nevertheless, this motor theory of learning does not take into consideration the salience of variability in practice while mastering a procedure.

With the advent of soft embalming techniques, cadavers are increasingly used for teaching procedural skills owing to their relatively higher fidelity, haptic parameters, and life-like simulation compared to mannequins and virtual reality simulator. On the flip side, many cadaver-based workshops suffer a lack of structuring and sophisticated feedback. Feedback from cadaveric skill workshops should be structured to provide feedback regarding the knowledge of results and knowledge of performance to aid the mastery of standards. In this context, we emphasize the importance of knowledge about the theories involved in motor learning, which ideally should be incorporated while designing surgical skill cadaveric workshops and in the absence of which procedural skill training might not effectively translate to the clinical environment. Supplementing the established framework with learning theories will suffice the long-term outcomes of the skill training sessions, which is reaching mastery standards in performing that particular procedure. 
